# Amfetamine and methylphenidate medications for attention-deficit/hyperactivity disorder: complementary treatment options

**DOI:** 10.1007/s00787-012-0286-5

**Published:** 2012-07-05

**Authors:** Paul Hodgkins, Monica Shaw, David Coghill, Lily Hechtman

**Affiliations:** 1 Shire Pharmaceuticals, 725 Chesterbrook Boulevard, Wayne, PA 19087 USA; 2 Norgine Pharmaceuticals, Uxbridge, UK; 3 Centre for Neuroscience Division of Medical Sciences, University of Dundee, Dundee, UK; 4 Division of Child Psychiatry, McGill University, McGill University Health Center, Montreal Children’s Hospital, Montreal, QC Canada

**Keywords:** Amfetamine, ADHD, Central nervous system stimulants, Medication adherence, Methylphenidate

## Abstract

Attention-deficit/hyperactivity disorder (ADHD) is one of the most common neurodevelopmental disorders among school-aged children. It is highly symptomatic and associated with significant impairment. This review examines the role of stimulant medications in the treatment of children and adolescents with ADHD. Published clinical studies that compared methylphenidate- and amfetamine-based stimulants in children and adolescents with ADHD support the therapeutic utility of stimulant treatments, and suggest robust efficacy and acceptable safety outcomes in groups treated with either stimulant. Evidence-based guidelines agree that each patient with ADHD is unique and individual treatment strategies that incorporate both drug and non-drug treatment options should be sought. In seeking to optimize individual response and outcomes to stimulant therapy, important considerations include the selection of stimulant class, the choice of long- or short-acting stimulant formulations, addressing effectively any emergent adverse effects and strategies aimed at enhancing adherence to dosing regimen and persistence on therapy.

## Introduction

Attention-deficit/hyperactivity disorder (ADHD) is one of the most common neurodevelopmental disorders among school-aged children, with a worldwide prevalence estimated to be 5.29 % (95 % confidence interval [CI], 5.01–5.56 %), based on a meta-analysis of 102 studies incorporating more than 170,000 participants from all continents [79]. ADHD is a heterogenous disorder that is, however, characterized by the core symptoms of inattention and hyperactivity/impulsivity [2]. ADHD results in significant impairment, and its treatment should address both the core symptoms and any comorbid conditions, behavioural or psychosocial impairments, and learning difficulties that may be present [17, 66, 75, 91].

This review examines the role of stimulant medications as part of a multimodal treatment strategy in children and adolescents with ADHD. The review first explores the place of stimulant medications in clinical treatment guidelines around the world and then briefly reviews the overlapping but distinct mechanisms of actions of the methylphenidate (MPH) and amfetamine (AMF) classes of stimulant in the pathophysiology of ADHD. Next, we provide an update of direct and indirect clinical comparisons of efficacy of these stimulants in the treatment of ADHD. Finally, we discuss the role of stimulants within a comprehensive strategy aimed at optimizing treatment for the benefit of an individual with ADHD and their family members or caregivers.

## Treatment recommendations in clinical guidelines

Guidelines from around the world differ in their treatment recommendations [85]. There is, however, general agreement that a comprehensive, multimodal treatment plan should be developed by the clinician, patient and family working closely together. In this plan, psychoeducation, parent/caregiver management training, behavioural and educational intervention, and medications are balanced to create the optimum treatment paradigm for each individual with ADHD [1, 15, 17, 66, 75, 77, 91, 93]. Specific treatment plans will be based, in part, on problems and impairments identified for the child and on access to and funding of healthcare resources. These differ by jurisdiction and geography, both between and within different countries.

Considerable evidence has accumulated over several decades that most patients with ADHD symptoms can be successfully treated by psychopharmacotherapies [33, 61] as part of a comprehensive treatment approach. Short- and long-acting formulations of the stimulants MPH and AMF, including the recently introduced AMF prodrug lisdexamfetamine dimesylate (LDX), the selective noradrenaline reuptake inhibitor atomoxetine, and the α_2_ adrenergic receptor agonists clonidine and guanfacine (including short- and long-acting formulations) are all approved by the US Food and Drug Administration (FDA). However, the range of ADHD medications available to patients and physicians is not as extensive in many countries outside North America. In the USA, the American Academy of Child and Adolescent Psychiatry Practice Parameters recommend that treatment plans consist of psychopharmacotherapy and/or behavioural therapy. The initial medication should be one of the following FDA-approved drugs: MPH, AMF, mixed amfetamine salts, or atomoxetine [1, 75, 77]. The American Academy of Pediatrics (AAP) recommends that pre-school children receive behavioural therapy, with MPH only prescribed if moderate-to-severe dysfunction remains. For school-aged children, the AAP recommends FDA-approved medications and/or behavioural therapy, and for adolescents recommends the use of medications and that behavioural therapy may be used. With regard to medications, the AAP considers the evidence of efficacy to be particularly strong for stimulants and less strong for atomoxetine, long-acting guanfacine and long-acting clonidine [1, 75, 77]. The noradrenaline and dopamine reuptake inhibitor bupropion and tricyclic antidepressants, including imipramine, are listed within US guidelines as medication options for ADHD, but are not approved [75]. In Australia, MPH and AMF are also both recommended as first-line treatments [93], and in Canada, long-acting preparations of MPH and AMF or atomoxetine are all considered to be the first-line treatments [17].

In European countries, pre-school children and school-age children with ADHD with moderate impairment, psychoeducation and behavioural intervention are generally recommended as first-line treatment. In cases of severe ADHD with severe impairment, of moderate impairment that has failed to respond to psychoeducation, and when behavioural interventions are unavailable, medication should be offered [66, 91]. MPH, in short- or long-acting formulations, is generally recommended as the first choice medication for ADHD in Europe. Atomoxetine, though generally less effective than stimulants, is also widely available and may be recommended as an alternative to MPH [9, 24, 57, 63, 66, 84, 91]. AMF formulations are less widely available, typically due to either not being approved by regulatory agencies or not being placed on national formularies. Unusually within Europe, both MPH and AMF classes of stimulant are approved in the UK and are covered in national guidelines. In England and Wales, treatment algorithms recommend MPH as the first-line medication for ADHD, with atomoxetine as a second-line option. Although short-acting AMF is approved for ADHD, the National Institute of Health and Clinical Excellence (NICE) considered that the published trials were not of good enough quality to be included in their review, and so AMF is recommended only when symptoms are unresponsive to the maximum tolerated dose of MPH or atomoxetine [66]. National recommendations are reflected by a marked imbalance in prescribing patterns for the two classes of stimulant for the treatment of ADHD, the numbers of prescriptions in England (2010 figures) for MPH and AMF were 661,463 and 45,519, respectively [65]. Even in Scotland, where short-acting AMF is considered as a potential second-line treatment, it is prescribed disproportionately less frequently than MPH. In addition to the stimulants and atomoxetine, clonidine, guanfacine, bupropion, modafinil and tricyclic antidepressants are listed within European guidelines, but are not approved, as medication options for ADHD [66, 91].

Long-acting stimulant formulations are as efficacious as their short-acting counterparts. In a meta-analysis of 32 clinical studies in children and adolescents (mean ages within studies ranged from 8 to 15 years), there was no difference in effect sizes for dependent measures (standardized mean difference [SMD]; 95 % CI) observed for all studies that investigated short-acting (0.99; 0.88–1.1) and long-acting stimulants (0.95; 0.85–1.1) [38]. Long-acting stimulants offer the advantages of not having to be taken during the school day, thereby reducing stigma for the patient and the logistical problems for the school of storing and administering scheduled medications. In addition, once-daily formulations result in enhanced compliance, more consistent and extended coverage throughout the day, and reduced abuse potential than short-acting formulations [9, 75]. Short-acting stimulants have the advantages of greater flexibility of dosing and lower cost. Guidelines recommend that individual clinical choice will determine whether long- or short-acting stimulant medications should be used [9]. However, some guidelines (Canada) recommend the use of long acting stimulants as first line [17].

## In vitro and in vivo pharmacologies of MPH and AMF

**Fig. 1:**
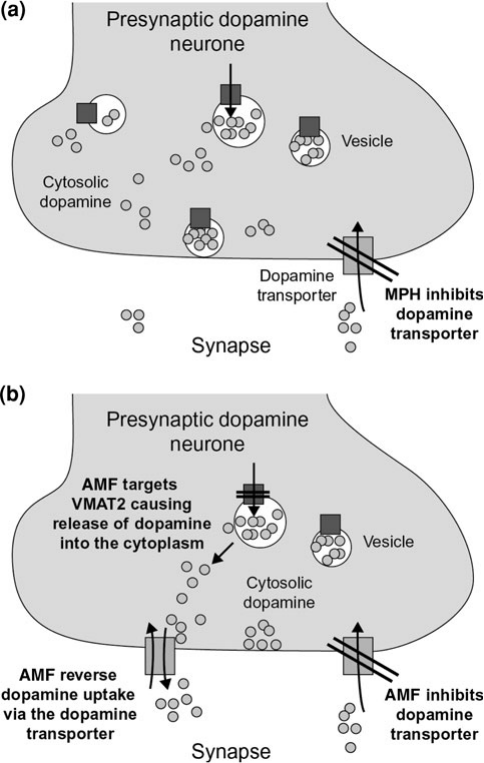
Overlapping but distinct putative mechanisms of action of **a** methylphenidate (*MPH*) and **b** amfetamine (*AMF*) at the dopamine synapse. *VMAT2* vesicular monoamine transporter 2

The aetiology of ADHD is complex, with multiple genetic and non-genetic factors implicated [22, 41]. However, recent evidence has converged to suggest that catecholamine neurotransmission is impaired in the brains of patients with ADHD [6, 7, 13]. Furthermore, stimulants and the non-stimulant atomoxetine increase synaptic catecholamine concentrations in the brain, particularly in the prefrontal cortex, although their precise mechanisms of action differ (Fig. 1).

The primary molecular targets of MPH are plasma membrane dopamine and noradrenaline transporters [60]. In vitro experiments demonstrate that uptake of dopamine and noradrenaline is inhibited by dl-threo-MPH with modest potency (inhibition constant [*K*_i_], 160–341 and 40–238 nM, respectively) [49]. Intraperitoneal administration of dl-threo-MPH 10 mg/kg to spontaneously hypertensive rats elicits a rapid 3–4-fold increase in extracellular concentrations of noradrenaline in the prefrontal cortex and dopamine in the striatum, peaking within 45 min of dosing, and remaining above control levels for at least 3 h [48].

Like MPH, d-AMF inhibits uptake of dopamine and noradrenaline with modest potency (*K*_i_ 34–225 and 39–55 nM, respectively). Unlike MPH, d-AMF also inhibits 5-hydroxytryptamine (5-HT) uptake (*K*_i_ 1.4–3.8 μM) [49]. d-AMF also induces the release of monoamines from presynaptic terminals [49], possibly via mechanisms that include an interaction with vesicular monoamine transporter 2, and the reversal of plasma membrane monoamine transporters [30, 52, 53, 82, 90]. Evidence of a weak affinity for monoamine oxidase (*K*_i_ 20 μM) suggests that d-AMF also inhibits the metabolism of monoamines [49]. Intraperitoneal administration of d-AMF 1 mg/kg to spontaneously hypertensive rats elicits a 15-fold increase in striatal dopamine concentrations 30 min post-dose that return to control levels within 90 min, and a fourfold increase in noradrenaline concentrations in the prefrontal cortex within 45 min of dosing that remain above control levels for at least 3 h. Unlike MPH, d-AMF also elicits elevations in extracellular 5-HT concentrations in vivo [55].

## Clinical comparisons of MPH and AMF

Table 1 presents a summary of the MPH- and AMF-based stimulants that are used in the treatment of ADHD. In clinical studies, the efficacy and side effects of a treatment in respect of a particular outcome measure may be assessed at the overall group mean or individual level (response rate). There are multiple methods for comparing the efficacy of ADHD medications. The strongest evidence is provided by direct, head-to-head, parallel-group studies. However, the paucity of such studies means that other clinical trial designs, including crossover studies and meta-analyses, must be used to compare efficacy across stimulant treatments.

**Table 1:** Structure of amfetamine (AMF) and methylphenidate (MPH), and examples of commercial formulations used in the treatment of ADHD

	Short-acting formulations			Long-acting formulations^a^		
	Drug	Duration of action (h)		Drug	Composition	Duration of action (h)
Amfetamine (AMF, International Non-Proprietary Name)						
	Adderall^®^ (mixed salts of dl-AMF)	4–6 [3]		Adderall XR^®^ (mixed salts of dl-AMF)	Capsulated biphasic beads	8–12 [3, 9, 17]
	Dexedrine^®^ (d-AMF sulphate)	4–6 [3, 17]		Dexedrine Spansule^®^ (d-AMF)	Capsulated biphasic beads	6–10 [3, 17]
	DextroStat^®^ (d-AMF sulphate)	4–6 [3]		Vyvanse™ (d-AMF)	Prodrug	13–14 [17]
Methylphenidate (MPH)						
	Focalin^®^ (d-MPH)	4 [3]		Biphentin^®^ (racemic MPH)	Capsulated biphasic beads	10–12 [17]
				Concerta^®^ (racemic MPH)	Osmotically controlled release	8–12 [3, 9, 17, 62]
	Medikinet^®^ (racemic MPH)	4 [3, 62]		Daytrana^®^ (racemic MPH)	Transdermal patch delivery system	8–12 [3]
	Metadate^®^ (racemic MPH)	4 [3, 62]		Equasym XL^®^/Metadate CD^®^ (racemic MPH)	Capsulated biphasic beads	8 [3, 9, 62]
	Methylin^®^ (racemic MPH)	4 [3, 62]		Focalin^®^ XR (d-MPH)	Capsulated biphasic beads	8–12 [3]
	Ritalin^®^ (racemic MPH)	4 [3, 62]		Metadate ER^®^ (racemic MPH)	Wax matrix tablets	8 [3, 62]
				Medikinate^®^ retard (racemic MPH)	Capsulated biphasic beads	7–8 [3, 9]
				Methylin^®^ ER (racemic MPH)	Wax matrix tablets	8 [3, 62]
				Methylphenidate SR^®^ (racemic MPH)	Wax matrix tablets	8 [3]
				Ritalin LA^®^ (racemic MPH)	Capsulated biphasic beads	8–10 [62]
				Ritalin SR^®^ (racemic MPH)	Wax matrix tablets	8 [3, 62]

*ADHD* attention-deficit/hyperactivity disorder, *d*-*AMF* dexamfetamine, *dl*-*AMF* racemic amfetamine, *MPH* methylphenidate

^a^Subdivided into intermediate and long-acting formulations by some authors [27, 75]

### Search strategy for the identification of published clinical comparisons of MPH and AMF

PubMed literature searches were conducted (in March 2011) for papers containing combinations of MPH-based studies (search terms: methylphenidate, Biphentin, Concerta, Daytrana, Equasym, Focalin, Medikinet, Metadate, Methylin, Ritalin) and AMF-based studies (search terms: amfetamine, Adderall, Dexedrine, Dextrostat, lisdexamfetamine). Results were limited to ‘clinical trial’ but were not limited by publication date. A total of 176 references were identified, of which 150 had English language abstracts. Identified papers were filtered for relevance based on the content of their abstracts. Inclusion criteria for references were the reporting of clinical outcome data (not biochemical, cytotoxicological, or preclinical data), concerned with ADHD and its treatment (not drug abuse, drug abuse liability, or comorbid symptoms such as tics), reported randomized, double-blind, controlled trials (not open label or *n*-of-1 trials), and reported comparisons of MPH- and AMF-based stimulants (not pooled stimulant groups or studies in which one or other stimulant was a simple positive control).

## Direct clinical comparisons of MPH and AMF

### Head-to-head comparisons

The results of the search for published randomized clinical studies that directly compare MPH- and AMF-based stimulants are shown in Table 2. Of the 13 published studies that were identified, there was one parallel-group (i.e. head-to-head) study [76]. This study compared short-acting mixed AMF salts (MAS) with short-acting MPH in the treatment of 58 children with ADHD. As the result of a dose optimization protocol designed to arrive at the ideal balance between efficacy and side-effects, mean daily doses of short-acting MAS and MPH in the final week of the study were 12.5 and 25.2 mg, respectively. The mean daily inattention/overactivity factor of the Inattention/Overactivity with Aggression (IOWA) Conners’ Teacher Rating Scale (CTRS) in the MAS-treated group (mean 0.49) was statistically superior to that of the MPH-treated group (mean 0.81), and both were statistically superior to placebo (mean 1.49). Similarly, the aggression/defiance factor of the IOWA CTRS in the MAS group (mean 0.29) was statistically superior to the MPH group (mean 0.49), and both were statistically superior to placebo (mean 0.72). Further, patients treated with MAS were superior to MPH in the Clinical Global Impressions-Improvements (CGI-I) scale (means 1.6 and 2.35 for the MAS- and MPH-treated patients, respectively) and both were superior to placebo (mean 3.22). The proportion of responders (i.e. improvement in CGI-I scores of 1 or 2) also favoured MAS treatment (MAS 90 %, MPH 65 %, and placebo 27 %) [76]. As will be discussed later, there was no statistical difference in parent-reported side-effects of moderate or severe intensity at the end of the study. Thus, evidence from this parallel-group comparison suggests the superiority of short-acting AMF- over short-acting MPH-based stimulants at optimized daily doses. The authors acknowledged, however, that the dose-optimization algorithm employed, may have limited dosing in the methylphenidate group [76]. It is also possible that the group differences could be a consequence of the longer half-life, and subsequent longer duration of action, of the MAS preparation (up to 6 h) compared to the MPH preparation (up to 4 h).

**Table 2:** Randomized clinical studies comparing treatment outcomes of methylphenidate and amfetamine formulations in ADHD

Reference	Study design	*n*	Mean age in years	Dose^a^	Efficacy	Tolerability	Response rate, *n* (%)			
							MPH	AMF	Either	Both
Winsberg et al. [99]	Crossover	18	8.5	Adjusted daily doses: MPH, ≤40 mg d-AMF, ≤60 mg	No difference in behavioural ratings between MPH and d-AMF	Insomnia and appetite loss No difference between treatments	NR	NR	14/18 (78)	11/18 (61)
Arnold et al. [5]	Crossover	29	8	Adjusted mean daily doses: MPH, 1.25 mg/kg d-AMF, 0.63 mg/kg Caffeine, 12.1 mg/kg	Both MPH and d-AMF superior to placebo Non-significant trend for AMF to be superior to MPH in all measures	Appetite loss for both drugs Stomach aches reduced in d-AMF vs. placebo	19/29 (66)	19/29 (66)	26/29 (90)	13/29 (45)
Borcherding et al. [12]	Crossover	18	9.6	Fixed escalating doses: MPH, 25–90 mg/day d-AMF, 10–45 mg/day	MPH produced greater lowering of motor activity than d-AMF		14/18 (78)	6/18 (33)	15/18 (83)	5/18 (28)
Pelham et al. [72]	Crossover	22	10.4	Fixed daily doses: MPH, 20 mg Ritalin SR-20^®^, 20 mg Dexedrine Spansule^®^, 10 mg Pemoline, 56.25 mg	In the context of an intensive summer treatment programme, dexedrine spansule and pemoline consistently had the most beneficial effects Treatment recommendations: Dexedrine Spansule^®^, 2/22; pemoline, 4/22; Ritalin SR-20, 4/22; MPH, 1/22; no medication (i.e. intense behavioural therapy) 7/22	Slightly more adverse events for Dexedrine Spansule^®^ than MPH or Ritalin SR-20^®^	NR	NR	15/22 (68)	NR
Elia et al. [35]	Crossover	48	8.6	Fixed escalating doses: MPH, 25–90 mg/day d-AMF, 10–45 mg/day	MPH produced greater reductions in motor activity than d-AMF Considerable individual differences in responses between the drugs observed	Insomnia and appetite disturbance were common for both drugs Both drugs were significantly worse than placebo for overly meticulous behaviour Nervous habits and mannerisms significantly worse than placebo for MPH only	38/48 (79)	42/48 (88)	46/48 (96)	34/48 (71)
Elia et al. [36]	Crossover	33	9.3	Fixed escalating doses: MPH, 25–90 mg/day d-AMF, 10–45 mg/day	Patients attempted more mathematics and reading tasks on either drug Percent correct for mathematics tasks improved for d-AMF only	Moderate, transient adverse events including appetite suppression and insomnia were common for both drugs	NR	NR	NR	NR
Efron et al. [31, 32]	Crossover	125	8.7	Fixed daily dose: MPH, 0.6 mg/kg d-AMF, 0.3 mg/kg	On Conners’ Teacher Rating Scale, response was superior for MPH for conduct problems and hyperactivity factors On parent-rating scale, MPH was superior for anxiety; 46 % chose MPH as the preferred drug, 37 % chose d-AMF as the preferred drug Only one non-responder to both drugs by three outcome measures	d-AMF caused appetite loss Insomnia and negative emotional symptoms worse for AMF than MPH	90/125 (72)	85/125 (68)	118/125 (94)	58/125 (46)
Pelham, Aronoff et al. [68]	Crossover	25	9.6	Fixed daily doses: Ritalin^®^, 10, 17.5 mg Adderall^®^, 7.5, 12.5 mg	In the context of an intensive summer treatment programme, the tested doses of Adderall^®^ produced greater improvement than those of Ritalin^®^ on many measures of behaviour in social and classroom settings The lower dose of Adderall^®^ was comparable with the higher dose of Ritalin^®^ Treatment recommendations: Adderall^®^, 7.5 mg, 11/25; 15 mg, 2/25; Adderall^®^ 7.5 mg or Ritalin^®^ 10 mg, 3/25; Ritalin^®^, 17.5 mg, 4/25, neither (i.e. intensive behavioural therapy only)	Averaged across all medication days, more evidence of loss of appetite and trouble sleeping for the high-dose Adderall^®^ treatment	16/25^b^ (64)	7/25^b^ (28)	21//25^b^ (84)	3/25^b^ (12)
Pelham, Gnagy et al. [69]	Crossover	21	10.3	Fixed daily doses: MPH, 0.9, 0.75, 0.3 mg/kg Adderall^®^, 0.6, 0.45, 0.3 mg/kg	In the context of an intensive summer treatment programme, total daily dose of 0.3 mg/kg/day Adderall^®^ equivalent to 0.6 mg/kg/day MPH in daily rates of behaviours in classroom and social settings. Treatment recommendations: MPH, 4/21, Adderall^®^, 6/21, 6/21 either drug, 5/21 neither (i.e. intensive behavioural therapy only)	Reports of appetite loss and trouble sleeping for both drugs when administered in the afternoon	10/21^b^ (48)	12/21^b^ (57)	16/21^b^ (76)	6/21^b^ (29)
Castellanos et al. [19]	Crossover	45	8.7	Fixed escalating daily doses: MPH, 25–90 mg d-AMF, 10–45 mg	MPH and d-AMF produced similar improvements in continuous performance test omission and commission errors		NR	NR	NR	NR
Sharp et al. [86]	Crossover	42	8.9	Adjusted daily doses: MPH, 10–70 mg d-AMF, 5–30 mg	Mean beneficial effects of MPH and d-AMF similar for all ratings	Mean adverse effects of MPH and d-AMF similar Loss of weight significantly greater for d-AMF than MPH	26/32 (81)	27/32 (84)	31/32 (97)	22/32 (69)
Pliszka et al. [76]	Parallel group	58	8.1	Adjusted mean daily doses: MPH, 25.2 mg Adderall^®^, 12.5 mg	Classroom inattentive and oppositional symptoms and CGI-I: both MPH and AMF superior to placebo Adderall^®^ significantly superior to MPH in teacher ratings and CGI-I Beneficial effects of Adderall^®^ persisted longer than MPH	Neither drug affected weight Greater number of patients on Adderall^®^ reporting stomach ache or sadness/tearfulness not significant after correction for multiple tests	13/20 (65)	18/20 (90)	NR	NR
Wilson et al. [98]	Crossover	35	17.5	Fixed daily doses: Concerta^®^, 72 mg Adderall XR^®^, 30 mg	Functional impairment composite score: Concerta^®^ and Adderall XR^®^ better than placebo; no difference between Concerta^®^ and Adderall XR^®^ Visual impairment and response inhibition: Concerta^®^ only better than placebo, no difference between Concerta^®^ and Adderall XR^®^		NR	NR	NR	NR

*ADHD* attention-deficit/hyperactivity disorder, *CGI*-*I* Clinical Global Impressions-Improvements, *d*-*AMF* dexamfetamine, *MPH* methylphenidate, *NR* not reported

^a^Dose was either fixed as a single dose, dose by body weight or dose escalation, or optimized based on efficacy and the emergence of adverse events

^b^Based on treatment recommendations at the end of the study

### Crossover studies

An advantage of crossover clinical trials is that the within-group design permits the comparison of the treatments in each individual patient, rather than at the group or population level only. A previous comparative review [4] of crossover studies of short-acting formulations of AMF and MPH found no consistent statistical differences in group means of outcome measures. Of a total of 174 patients in six crossover studies, 48 (28 %) responded better to AMF and 27 (16 %) responded better to MPH, and at least 72 (41 %) responded to both; in all studies except for one in which patients exhibited comorbid Tourette’s syndrome, there was a non-significant trend for superior response in patients treated with AMF over those treated with MPH [4].

Most of the crossover studies listed in Table 2 that compared the efficacy of MPH and AMF in patients with ADHD reported equivalence in outcome measures for the two classes of stimulant at the level of the group mean. Those studies that observed statistical superiority of one stimulant over the other for particular outcome measures are reviewed below.

Studies that reported outcomes that favoured MPH included an Australian study, in which 125 treatment-naïve children (aged 5–15 years) were randomly assigned to receive either MPH or AMF [31]. Doses were fixed and based on body weight (short-acting MPH 0.3 mg/kg twice daily; short-acting AMF 0.15 mg/kg twice daily). After 2 weeks of treatment, both stimulants induced significant improvements in baseline scores for all factors of the Conners’ Teacher Rating Scale-Revised (CTRS-R) and the Conners’ Parent Rating Scale-Revised (CPRS-R). In the CTRS-R, there was a statistically significant difference (MPH effect minus AMF effect) in favour of MPH in treatment-induced improvements in conduct problems (difference 3.31, 95 % CI 1.11–5.50, *p* < 0.01), hyperactivity factor (difference 2.78, 95 % CI 0.70–4.86, *p* < 0.01), inattentive-passive factor (difference 1.61, 95 % CI 0.30–2.92, *p* = 0.02) and hyperactivity index (difference 2.60, 95 % CI 0.69–4.51, *p* < 0.01). In the CPRS-R, the difference in improvement in favour of MPH reached statistical significance for the anxiety factor only (difference 1.20, 95 % CI 0.19–2.20, *p* = 0.02) [31]. In another study in which short-acting stimulant formulations of MPH 0.45–1.25 mg/kg and AMF 0.2–0.6 mg/kg were administered at breakfast and lunchtime to 18 boys (mean age 9.6 years), both stimulants significantly reduced motor activity (truncal activity counts per hour) compared with placebo, but the reduction was greater for MPH than for AMF between the hours of 11 a.m. and 1 p.m. [12]. A recent crossover study compared the effects of the long-acting stimulant formulations osmotic release oral system MPH (OROS MPH; maximum daily dose 72 mg) and extended-release MAS (maximum daily dose 30 mg) on neuropsychological functioning in adolescents with ADHD (*n* = 35; mean age 17.5 years) [98]. There were no significant differences between OROS MPH and extended-release MAS in any of the outcomes studied. However, OROS MPH, but not extended-release MAS, was statistically superior to placebo in distracter errors and distracter reaction time in the Go/No-Go test and in Recall Accuracy in the Delayed Matching-to-Sample test, perhaps reflecting greater than a twofold MPH to AMF dose-ratio than that considered to be equivalent [98, 101].

In contrast to the above results favouring MPH, responses to short-acting AMF (maximum daily dose 45 mg) were superior to short-acting MPH (maximum daily dose 90 mg) in a crossover study of classroom performance in boys aged 6–12 years with ADHD. Compared with placebo, both drugs produced statistically significant improvements in performance (percent correct responses) and number of problems attempted for reading tasks, and both drugs improved the number of attempted arithmetic problems. However, improved performance in arithmetic problems compared with placebo was statistically significant for AMF only (mean [standard deviation] percent correct: AMF 97.1 [4.6]; MPH 96.2 [5.6]; placebo 94.0 [7.9]) [36]. Again, the longer duration action of the amphetamine may have influenced these results.

### Meta-analyses

**Fig. 2:**
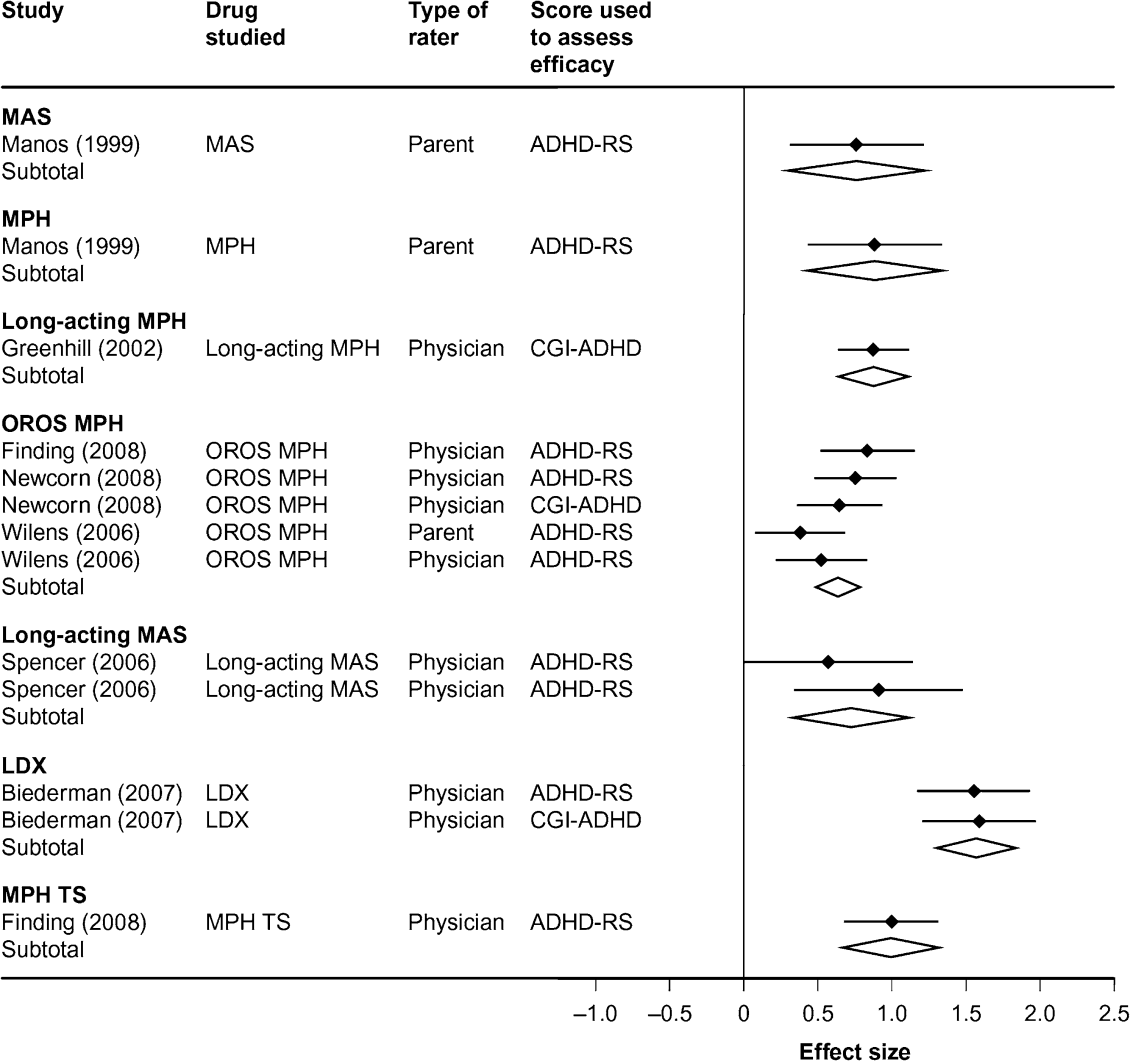
Effect sizes and confidence intervals for ADHD-RS and CGI outcomes in children. *Black diamonds* and *horizontal black lines* represent standardized mean difference effect sizes and 95 % confidence intervals, respectively. Pooled results are depicted as *open diamonds* with the effect size in the *centre of the diamond* and the 95 % confidence intervals depicted by the *left and right extremities of the diamond*. *ADHD*-*RS* attention-deficit/hyperactivity disorder rating scale, *CGI* clinical global impressions of ADHD severity, *LDX* lisdexamfetamine dimesylate, *MAS* mixed amfetamine salts, *MPH* methylphenidate, *OROS* osmotic release oral system*, TS* transdermal system. Figure adapted from Faraone [37], with permission

Using standardized effect sizes, it is possible indirectly to compare efficacy outcomes for particular treatments across studies. Standardized mean difference (SMD) is one way of calculating effect size. For example, an SMD of 1 indicates that the mean outcomes in drug and placebo groups differ by 1 (pooled) standard deviation. In interpreting SMD values, an SMD of 0.2 is considered small, 0.5 is considered medium, and ≥0.8 is considered large [21] (Fig. 2).

A series of recent meta-analyses have examined effect sizes of various outcome measures in patients with ADHD of different ages treated with different ADHD medications [38–40]. In a meta-analysis of 32 double-blind, placebo-controlled trials in youths (aged 6–18 years) with ADHD, meta-analysis regression found that effect sizes for non-stimulant medications (SMD = 0.57) were significantly smaller than for both short-acting (SMD = 0.99, F_1,31_ = 25, *p* < 0.0001) and long-acting stimulants (SMD = 0.95, F_1,31_ = 15, *p* < 0.0001) [38]. Although this analysis did not stratify stimulant therapies according to their MPH or AMF class, it is interesting to note that the largest effect sizes for both short-acting (MAS, SMD = 1.34) and long-acting (LDX, SMD = 1.52) stimulants were seen for AMF-based therapies. When effect sizes were compared across 23 randomized, controlled trials of 11 different short- and long-acting stimulants in children and adolescents with ADHD, robust drug effects were observed in all individual studies. Furthermore, across all of these studies, effect sizes for AMF products were significantly greater than for MPH products (SMD = 1.03 vs. 0.77, *t*_19_ = 2.5, *p* = 0.02). Regression analyses found that several study variables, including stimulant formulation (short-acting vs. long-acting), type of dosing (fixed vs. optimized), and study design (parallel vs. crossover), were not associated with SMD. It is, of course, possible that heterogeneity between study variables may have obscured possible associations between these variables and SMD. Nevertheless, three study design features were identified that they were associated with SMD (age, type of score [outcome or change], and rater [physician, parent, teacher, or patient]), but the finding that effect sizes for AMF were modestly greater than those for MPH held after correcting for these confounding variables [39]. Equivalence of dosing between AMF and MPH stimulants was not demonstrated. Using the method of numbers needed to treat, another way of comparing outcomes from different studies, the authors calculated that 2.0 patients were needed to be treated with AMF for each positive outcome for total ADHD symptoms compared with 2.6 patients for MPH [39]. Overall, these pooled analyses provide strong evidence for the efficacy of stimulant medications in ADHD, while one meta-analysis provides evidence of the greater efficacy of AMF-based drugs compared with MPH-based drugs in children and adolescents. Again, the longer duration action of short-acting AMF than short-acting MPH may have contributed to these results.

### Summary of clinical comparisons

Overall, some individual studies have demonstrated superiority of MPH over AMF, some have found superiority of AMF over MPH, and others have shown no differences between the two types of medications. When meta-analyses are performed summarizing all available evidence, the effect sizes observed with AMF are greater than those observed with MPH, although issues of comparable dosing and differences in the duration of action of short-acting stimulants should be taken into account when interpreting these data. However, given the currently available evidence, it has not been demonstrated that one stimulant is more efficacious than another at a population level; direct head-to-head studies would be required to establish this definitively.

## Tolerability of stimulant medications

There is considerable overlap in the adverse event profiles of MPH- and AMF-based ADHD medications [46, 47]. In the parallel-group comparison of MPH and MAS in children with ADHD, the adverse events reported in more than 10 % of patients in the MPH treatment group were tiredness, appetite loss, irritability, and anxiousness; in the MAS group they were stomach ache, irritability, negative emotion (sadness, tearfulness), appetite loss, tiredness, and headache [76]. The incidences of stomach ache and negative emotion were numerically greater in the MAS group than in the MPH group but the differences were not significant after applying the Bonferroni correction for multiple statistical tests [76].

In crossover studies, insomnia and appetite suppression were generally reported to be the most common adverse events for both classes of stimulant [46, 47]. The severities (but not frequencies) of insomnia, irritability, proneness to crying, anxiousness, sadness, unhappiness, and nightmares were all reported to be greater in children treated with AMF 0.15 mg/kg twice daily than with MPH 0.3 mg/kg twice daily [32]. The incidence of overall adverse events has been reported to be numerically greater in children receiving long-acting AMF 10 mg/day than in those receiving short-acting MPH 10 mg twice daily or long-acting MPH 20 mg/day [72]. Furthermore, in a comparison of the safety and tolerability of short-acting MPH 10 and 17.5 mg twice daily and MAS 7.5 and 12.5 mg twice daily, parent-rated incidences of moderate-to-severe (on average) trouble sleeping and loss of appetite were greater in high-dose, MAS-treated patients (trouble sleeping 12 %, loss of appetite 24 %) than in high-dose, MPH-treated patients (trouble sleeping 4 %, loss of appetite 4 %) [68]. Finally, over 3 weeks’ dosing, mean weight loss was significantly greater than placebo in girls treated with AMF (maximum dose 0.64 mg/kg twice daily, mean [standard deviation] change from baseline −1.1 kg [1.0 kg], *p* < 0.01) but not MPH (maximum dose 1.28 mg/kg twice daily, mean [standard deviation] change from baseline −0.4 kg [1.1 kg], not significant) [86].

In contrast, nervous habits and mannerisms have been reported as being more common in boys treated with MPH (maximum mean dose 2.5 mg/kg twice daily), but not AMF (maximum mean dose 1.3 mg/kg twice daily), than in those treated with placebo [35]. In addition, the incidence of stomach aches in children was lower with AMF (mean dose 18.5 mg/day), but not MPH (mean dose 37.9 mg/day), than with placebo [5].

Based on the reviewed studies, the adverse event profiles of the two classes of stimulants appear to be similar. Some studies suggest that the frequency and severity of adverse events may be somewhat greater with AMF than with MPH products when AMF is compared with MPH, whereas side effects with MPH may be more common than with AMF when both drugs are compared with placebo.

## Optimizing medication for an individual

The aim of optimizing an individual’s medication strategy is to achieve the maximum reduction of symptoms, or even the remission of ADHD, without the appearance of intolerable side effects. While it used to be the case that improvement in symptoms was the primary goal of treatment, clinicians are increasingly seeking treatment strategies that result in remission of ADHD in an individual. By remission we mean the loss of diagnostic status, with minimal or no symptoms, and optimal functioning with minimal impairment [88]. The likelihood of the appearance of particular side effects differs between individuals and between each medication class [46, 47]. Evidence-based guidelines recognize that each patient is unique and that treatment strategies should be tailored to an individual’s situation, taking into account a broad range of factors including age, type of ADHD, comorbid symptoms, treatment history, and the attitudes of patients and parents/caregivers to ADHD medications. The initial selection of medication strategy for an individual requires consideration of the class of drug, dose and desired pharmacokinetic properties (including speed of onset and duration of action). Subsequent optimization of treatment involves on-going assessment for adequate efficacy and remission of impairment, and monitoring and treating treatment-emergent adverse events and adherence to the agreed therapeutic regimen [1, 15, 17, 66, 75, 77, 91, 93].

### Choice of stimulant class

Although group average responses to MPH and AMF in patients with ADHD are similar, individuals may respond very differently to the two drugs. While approximately two-thirds of patients typically experience improvements in various symptom domains in response to a single stimulant, if patients with an unsatisfactory response try the alternative class of stimulant, the proportion of patients who respond to one of the drugs may be as high as 95 % [4, 31]. Updated response data for studies directly comparing MPH- and AMF-based stimulants are presented in Table 2. Of eight studies containing data for MPH and AMF formulations, the proportion of responders was higher for AMF in four studies, higher for MPH in three studies, and were equivalent in one study. In the only head-to-head comparison of stimulants, 90 % of patients responded to MAS, 65 % responded to MPH, and 27 % responded to placebo [76]. When numbers of responders for each stimulant were combined across studies, 226 of 318 patients (71 %) responded to MPH and 216 (68 %) responded to AMF, suggesting that there is no meaningful difference in numbers of responders for MPH and AMF in ADHD. However, the proportion of patients responding to either class of stimulant (287 of 316 patients 91 %) was higher than those responding to each single stimulant.

These analyses confirm previous assertions that non-response is uncommon when an individual is offered both a MPH and an AMF [35], and that responses to the two classes of stimulant, although similar in the overall ADHD population, does vary between individuals [4]. Differences in the metabolic pathways and mechanisms of action of MPH and AMF (see above), the genotype of an individual [44, 89] and the pathophysiology of their ADHD [26] may be important factors in determining an individual’s response to the different stimulant drugs. In terms of clinical practice, these differential response rates support clinical guidelines that recommend that MPH and AMF are equally valid first-choice medications for the treatment of ADHD, and that if the first-selected stimulant class proves to be unsatisfactory, then a stimulant from the second class should be tried [75, 77].

The selection of which stimulant class to start with may be aided by methods for identifying patient subgroups that may preferentially respond to medication, including pre-specified and post-hoc subgroup analyses of clinical data [11, 23, 28, 29, 54, 87]. However, there are well-accepted constraints of both a priori and post-hoc specification [8, 56, 74, 78]. These limitations are starting to be addressed by the development of personalized treatment selection techniques; these combine patient characteristics from clinical trial data to form a risk score and then performing analyses on subgroups using a two-stage process that allows for treatment responses at an individual level to be made [16, 94, 102]. These methodologies have the advantage that they can systematically use multivariate regression models to select and combine multiple baseline covariates from different levels of analysis to define subgroups. With a view to the future, pharmacogenomics and non-genetic biomarkers may assist in the optimization and individualization of ADHD pharmacotherapy, although this is not yet possible [10, 19, 34, 44, 50, 89].

### Choice of stimulant formulation

Reductions in the symptoms of ADHD by stimulants depend upon achieving sufficient occupancy of their molecular target(s) in the brain [95]. Positron emission studies suggest that peak occupancy of the dopamine transporter is reached approximately 60 min after oral dosing with short-acting MPH [96]. However, the elimination half-life of short-acting MPH is reported to be approximately 3 h [51] and of AMF to be approximately 7 h [14]. Therefore, two or, in the case of short-acting MPH, three daily doses are necessary to maintain therapeutic concentrations of stimulants within the brain throughout the day. For children and adolescents, repeat dosing may be undesirable because of the difficulties associated with storing and administering scheduled drugs within a school environment, the stigma associated with receiving medication during the school day, fragmented coverage with multiple short-acting doses, the potential for diversion of drug and the possible impact on adherence to the dosing regimen [100].

To extend the duration of action (i.e. symptomatic control) of stimulant medications, several long-acting formulations of MPH and AMF have been introduced that extend the pharmacokinetic and pharmacodynamic profile of the drugs (Table 1) [43, 61]. Most formulations depend on the slow, sustained release of the active ingredient in the stomach via the use of technologies such as wax matrix tablets, capsulated biphasic beads, or osmotically controlled release systems. Long-acting formulations mean that systemic exposure, and hence efficacy (symptom control), is maintained for longer periods, resulting in correspondingly improved convenience, confidentiality and compliance, more consistent coverage, and reduced abuse potential [43, 61, 75]. LDX is the first stimulant to use prodrug technology to modify the delivery profile. In its parent form, LDX is inactive and requires enzymatic cleavage in the blood to yield AMF. The combination of short- and long-acting formulations provides a range of treatment options lasting from approximately 4 h to more than 12 h. The demonstration that the efficacy of LDX in children is maintained for at least 13 h [97], suggests that this prodrug is the longest-acting stimulant formulation [25, 45]. However, since the drug needs to be absorbed and then cleaved in the bloodstream before it can be active, the onset of action may be somewhat delayed and may occur 1.5–2 h after ingestion [97]. In selecting an ADHD medication, stimulants may be contraindicated or the patient or caregivers may express a preference for non-stimulants. In such cases, a non-stimulant such as atomoxetine may be considered. Generally, the non-stimulants are considered less effective than the stimulants. Where stimulants are considered appropriate, the choice of short- or long-acting stimulant should be based on both clinical requirements and the preferences of an individual and their family [9].

### Adherence to dosing regimen and persistence on therapy

Despite the carefully managed nature of the Multimodal Treatment Study of Children with ADHD (MTA), saliva assays for MPH revealed that approximately 25 % of 254 patients in the medication arms of the study were non-adherent on 50 % or more of repeated assays, and that barely half (54 %) were adherent at every assay point [67]. Furthermore, discrepancies were uncovered between parents’ reports of adherence and the outcomes of the saliva assays [67]. These results indicate that there is considerable potential to improve pharmacotherapy outcomes by improving adherence. Several strands of evidence suggest that medication adherence may be improved by tailoring the selection of drug to an individual patient. Discrete choice experiments suggest that long-acting stimulants, with consistent therapeutic coverage throughout the day, are the preferred stimulant formulations of most patients [20, 42, 58, 64], and retrospective claims analyses suggest that long-acting stimulants are generally associated with enhanced adherence and persistence in patients of all ages compared with short-acting stimulants [20, 59, 80, 83]. In addition to drug regimens that patients find convenient, other strategies to improve adherence include improved communication between physicians, caregivers, and patients; clear instruction and encouragement; peer support groups; advice about reminders to take medication and incorporating medication into daily routines; and the use of positive reinforcement to improve attitudes to medication [66]. Addressing adverse events effectively may also contribute to promoting adherence.

### Multimodal treatment

The importance of utilizing a multimodal treatment strategy that incorporates both medication and non-drug interventions is recognized by all ADHD clinical guidelines [1, 17, 66, 75, 91]. Indeed, as mentioned previously, non-drug interventions are the first-line treatment in school-aged children and adolescents with moderate ADHD and moderate impairment in many European countries [66, 91]. Behavioural therapy alone can produce improvements in ADHD compared with baseline [73]. In the MTA, for example, both medication and intensive behavioural therapy provided superior treatment outcomes to treatment in the community, even though the community treatment often included medication [92]. As the medication arm of the MTA was superior to both the behavioural and community treatment arms, these data provide further support for the notion that for medication treatments to achieve optimal effectiveness they need to be carefully titrated and monitored. The MTA also demonstrated that there were benefits in combining medication and behavioural therapy in certain non-core ADHD symptom domains (including aggression, internalizing symptoms, social skills, and parent–child relations), compared with either treatment approach alone [92]. Furthermore, non-adherence resulted in greater deleterious effects in the medication management group than in the combined treatment group [67]. The results from the MTA study confirmed those of earlier studies in children attending summer treatment programmes that demonstrated the reinforcing and synergistic outcomes of pharmacotherapy and behavioural therapy [18, 70, 71]. Together, these data illustrate the potential therapeutic advantages of combining intensive behavioural therapy with carefully crafted medication management [81].

## Conclusions

Randomized and controlled clinical trials indicate that MPH and AMF offer robust medication options for the treatment of ADHD. In the drive to improve the treatment of ADHD for an individual, the nature of the stimulant, its formulation and optimization of the dosing regimen, with careful on-going monitoring of both positive and negative medication effects and adherence are all important considerations. Furthermore, concurrent non-drug treatments, including behavioural therapy, should all be considered as part of a multimodal treatment strategy. For those patients or caregivers who prefer not to take stimulants or for whom stimulants are contraindicated, non-stimulant drug options are available, though generally less effective.

## Abbreviations

ADHD: Attention-deficit/hyperactivity disorder
AMF: Amfetamine
CGI-I: Clinical global impressions-improvements
CI: Confidence interval
CPRS-R: Conners’ Parent Rating Scale-Revised
CTRS: Conners’ Teacher Rating Scale
CTRS-R: Conners’ Teacher Rating Scale-Revised
d-AMF: Dexamfetamine
5-HT: 5-Hydroxytryptamine
IOWA: Inattention/overactivity with aggression
LDX: Lisdexamfetamine dimesylate
MAS: Mixed amfetamine salts
MPH: Methylphenidate
MTA: Multimodal treatment study of children with ADHD
OROS MPH: Osmotic release oral system methylphenidate
SD: Standard deviation
SH: Spontaneously hypertensive
SMD: Standardized mean difference
